# Pilot implementation projects in low- and middle-income countries to guide surgical quality improvement using best practice recommendations

**DOI:** 10.3389/frhs.2025.1423429

**Published:** 2025-06-17

**Authors:** Lye-Yeng Wong, Saad Hussain, Michael Labib, Richard Henker, Chizoba Efobi, Ndubuisi Mokogwu, Jeremiah Agbons Igunma, Seye Mesfine Minas, Tsegazeab Laeke, Mark Ferguson, Cheng Har Yip, Andrew Hill, Jaymie Henry

**Affiliations:** ^1^ Department of Cardiothoracic Surgery, Stanford University, Palo Alto, CA, United States; ^2^ Institute of Global Surgery, Royal College of Surgeons Ireland, Dublin, Ireland; ^3^ Department of Anesthesia, University of Pittsburgh, Pittsburgh, PA, United States; ^4^ Department of Surgery, University of Benin Teaching Hospital, Benin City, Nigeria; ^5^ Department of Public Health and Community Medicine, University of Benin Teaching Hospital, Benin City, Nigeria; ^6^ Department of Medical Microbiology, University of Benin Teaching Hospital, Benin City, Nigeria; ^7^ Department of Surgery, Ayder Comprehensive Specialized Hospital, Mekele, Ethiopia; ^8^ Department of Neurosurgery, Addis Ababa University, Addis Ababa, Ethiopia; ^9^ Department of Cardiothoracic Surgery, University of Chicago, Chicago, IL, United States; ^10^ Department of Surgery, University of Malaya, Kuala Lumpur, Malaysia; ^11^ Department of Surgery, The University of Auckland, Middlemore Hospital, Auckland, New Zealand; ^12^ Department of Cardiothoracic Surgery, Baylor College of Medicine, Houston, TX, United States

**Keywords:** global surgery, best practices, implementation, surgical quality improvement, low- and middle-income countries (LMICs)

## Abstract

**Background:**

Adherence to Best Practice Recommendations (BPRs) has been shown to improve morbidity and mortality in surgical healthcare delivery in low and middle-income countries (LMICs).

**Methodology:**

Three LMIC healthcare centres in Laos, Nigeria, and Ethiopia were chosen to participate in the implementation pilots through existing cross-collaborative partnerships. Local teams were assembled to conduct needs assessment analyses prior to implementation study design. The projects are ongoing, and preliminary results are presented using descriptive analysis.

**Results:**

The BPRs chosen for each site were: hand hygiene in Lao PDR, antimicrobial stewardship in Nigeria, and trauma in Ethiopia. The World Health Organization (WHO) hand hygiene observation tool was used to determine baseline hand hygiene compliance in a children's hospital in Lao People Democratic Republic (PDR), revealing that 56.1% of hand hygiene opportunities were missed. A gap analysis was conducted in an academic Nigerian hospital to investigate antibiotic use in surgical patients, which found that 81.2% of antibiotic use was for prophylactic vs. empiric indications. Lastly, the emergency medical technician national curriculum as set by the Ethiopian Ministry of Health was reviewed by local experts and a 15-module supplemental curriculum was developed to include additional topics such as managing large-scale events, transport of emergency patients, advanced life support, and establishing quality standards.

**Conclusion:**

Through international collaboration spearheaded by local stakeholders, we initiated baseline needs assessments in 3 countries to identify pillars on which to build-up implementation projects based on BPRs. These scalable pilot projects can be used as a framework to promote further optimization and standardization of safe and quality surgical care in LMICs.

## Background

Surgery is an essential part of universal health coverage and there is a rapid need to strengthen surgical systems globally (1, 2). As global surgery has evolved from international health and tropical medicine into an academic discipline, approaches have also shifted (3). There is increased emphasis on sustainable partnerships, capacity building, and country-led initiatives rather than direct delivery of care by foreign organizations (4). Work remains to build global surgery as an equitable, effective field addressing disparities in surgical care worldwide (5, 6).

However, many LMICs face significant barriers to surgical quality improvement, including chronic underfunding of health systems, inadequate physical infrastructure (e.g., operating rooms, recovery wards, sterilization facilities), workforce shortages in both surgical and anesthesia care providers, and limited access to essential supplies and equipment (2, 5). Additionally, weak data collection and monitoring systems make it challenging to assess baseline performance and track improvements (4). These systemic constraints highlight the need for adaptable and context-driven implementation of best practice recommendations (BPRs).

Best practice recommendations (BPRs) are a method to standardize care and provide flexible guidelines to provide quality surgical care (7). There is increasing focus on identifying, implementing, and evaluating best practices to improve quality, safety, and ethics in global surgery initiatives (8, 9). In response, the G4 Alliance and the International Society of Surgery (ISS/SIC), two non-governmental organizations, established the International Standards and Guidelines for Quality Safe Surgery and Anesthesia (ISG-QSSA) Working Group. The objective of the working group was to discuss guidelines for surgical, anesthesia, trauma, and obstetric procedures, employing prioritization and distribution methods to propose changes from both a top-down and bottom-up perspective.

In 2021, the ISG-QSSA Working Group conducted three systematic reviews to generate evidence-based recommendations following the WHO Handbook for Guidelines Development (10–12). Eleven BPRs covering three topics of interest: (1) reducing surgical site infections, (2) improving quality of trauma systems, and (3) interventions to reduce maternal and perinatal mortality, were endorsed by the ISG-QSSA and then published by Henry et al. (7) With the next step of the process to achieving surgical excellence being pilot implementation of the aforementioned BPRs, the aim of this study is to trial the BPRs in various different institutions in low- and middle-income settings. By establishing validity evidence in the real-world context, we hope to demonstrate feasibility of scaling the 11 BPRs.

## Methods

### Participants and settings

The 11 BPRs were formally introduced to the global surgery community at the biannual International Surgical Week hosted by the International Society of Surgery, which occurred in Austria in August 2022. A call to participation was conducted at that time to low- and middle-income institutions that had interest in piloting one of the proposed BPRs as described above. There were minimal inclusion criteria except for confirmed local stakeholder buy-in from the requesting institution. Stakeholder buy-in was defined as formal endorsement from institutional leadership, commitment from frontline clinical staff to participate in the implementation process, and willingness to allocate internal resources such as personnel time, meeting space, and local data collection. This buy-in was assessed through preliminary engagement meetings and documented confirmation from institutional representatives prior to project initiation. There were no exclusion criteria regarding location or type of healthcare facility. From this call to participation, three projects were confirmed, including a surgical hand hygiene program in a children's hospital in Laos, a surgical antimicrobial stewardship program in an academic hospital in Nigeria, and a prehospital trauma program in a tier of hospitals in Ethiopia.

### Implementation strategies and outcome measures

The focus of this manuscript is to highlight the foundational work required to organize a pilot implementation project in a low- and middle-income setting. This study is a descriptive analysis, aimed at documenting the early stages of these pilot projects rather than assessing final outcomes. The results described are secondary to pre-implementation strategies including gathering stakeholder buy-in, creating a team of local champions aided by international counterparts, performing a baseline assessment, and identifying the exact intervention to be enacted. As the scope of each of the three proposed projects was very different, each project was conducted at its own appropriate speed, leading to variable levels of progress over the agreed upon 1-year time period. The pilots are thus still in progress, and the findings presented here provide an overview of the setup, initial challenges, and future directions. Each pilot site identified context-specific outcome measures to guide future evaluation, even though full data collection and analysis are ongoing.

**Figure 1:**
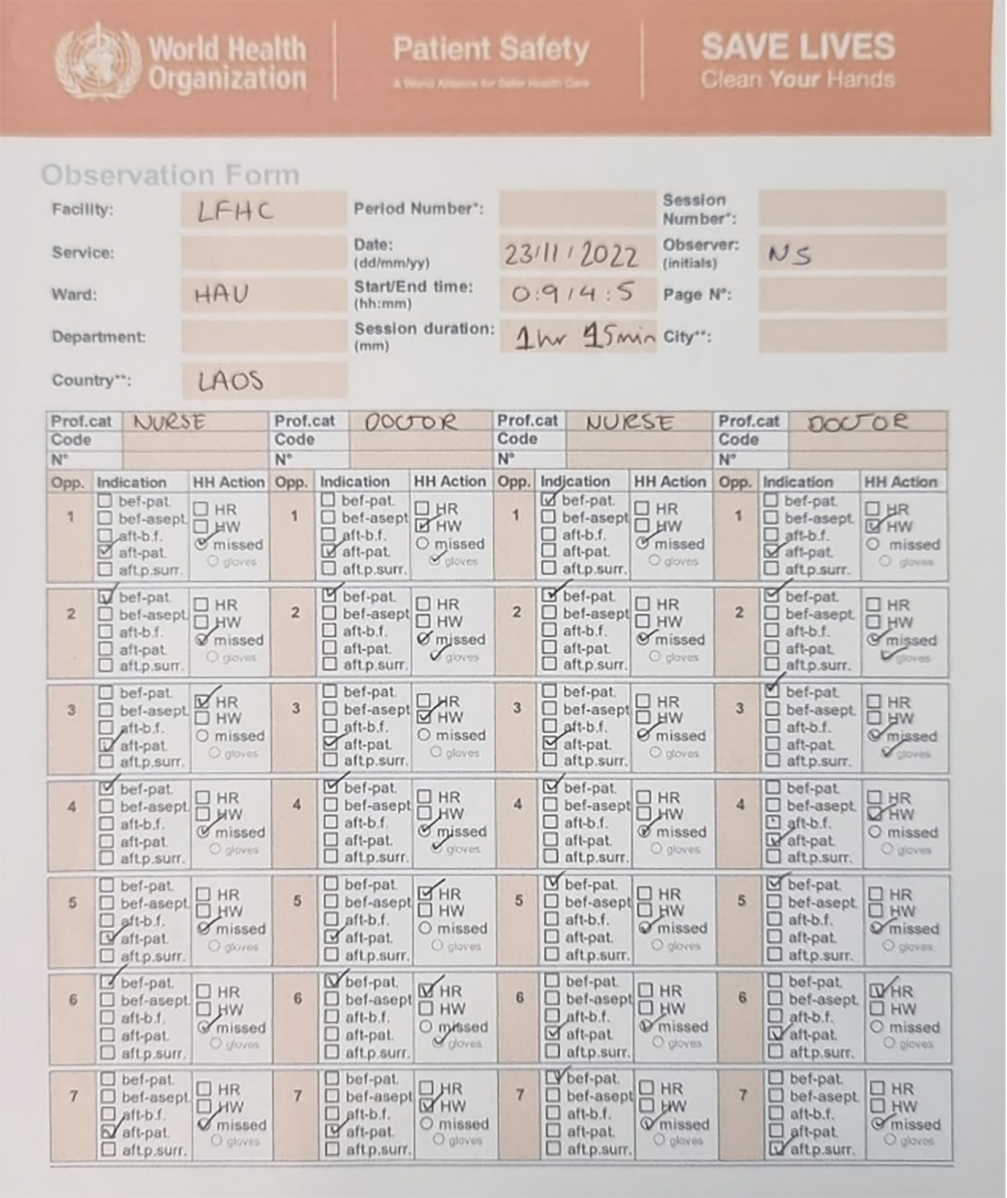
Data collection tool used to measure baseline hand hygiene compliance at Laos friends hospital for children.

For **Lao**, hand hygiene compliance will be measured at 12-monthly time points using the WHO Hand Hygiene Observation Form. This was adapted by the Laotian team and 5 observers were trained to use it as the main data collection tool (Figure 1). Outcome measures include the proportion of missed hand hygiene opportunities, stratified by provider type, unit, and WHO moment of care. Hand hygiene assessments were conducted at pre-specified intervals to track outcomes and determine the sustainability of this program.

For **Nigeria**, outcome measures will include antibiotic prescribing patterns, adherence to surgical antibiotic prophylaxis guidelines, and surgical site infection (SSI) rates. Prophylactic vs. empirical antibiotic use was defined using WHO and CDC guidelines, adapted to local protocols. Prophylactic use was defined as antibiotic administration within 60 min before incision in patients without signs of infection, while empirical use referred to antibiotic initiation based on clinical suspicion. Defined Daily Doses (DDDs) are under consideration for future analysis.

For **Ethiopia**, intended outcomes include pre- and post-training knowledge assessments of EMTs, as well as patient-centered indicators such as time to hospital arrival, stabilization success during transport, and care quality upon arrival. These will help assess the impact of the updated EMT curriculum and training model.

Lastly, during the post-implementation period, each project team will conduct debrief sessions to highlight strengths and weaknesses of the pilot and to create a plan for future measurement of long-term adherence to the proposed intervention.

### Statistical analysis

Data for the gap analysis was collected manually on paper at each institution and transferred to Microsoft Excel for review and analysis. Counts were tallied and reported as numbers and percentages. Statistical analysis was minimal, and results were descriptively summarized.

## Results

### Lao hand hygiene pilot: baseline assessment

Lao Friends Hospital for Children is a pediatric hospital opened in 2015 sustained by mostly local and a few expatriat staff. The G4 Alliance ISS-QSA working group partnered with this hospital in the region of Luang Prabang to help implement a surgical-focused hand hygiene program to improve adherence rates and create an adaptable framework that can be adopted in the adjacent provincial hospital and other healthcare centers in the region.

**Figure 2:**
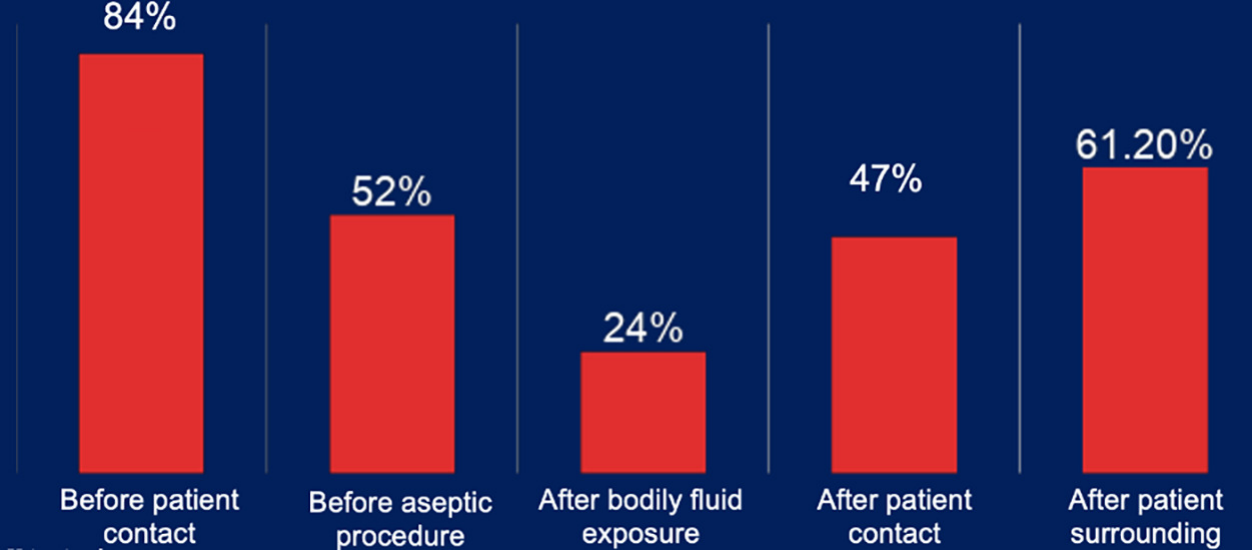
Missed hand hygiene occurrences stratified by the WHO 5 essential moments of hand hygiene.

Baseline assessments at the hospital included 329 hand hygiene opportunities observed across 51 participants, there are 56.1% of missed hand hygiene opportunities as defined by the WHO 5 essential moments of hand hygiene. The missed indications are primarily centered around moments before patient contact and before aseptic procedures, as compared to after patient contact, patient surroundings, and bodily fluid exposure (Figure 2). Preliminary data demonstrated that these high rates are equally rampant among all departments of the hospital, including the inpatient ward, neonatal intensive care unit, high acuity unit, and operating theaters; and that the missed opportunities are shared by both doctors and nurses equally.

Following the baseline assessment, the hospital implemented a multi-pronged awareness and education campaign using official materials from the WHO Hand Hygiene Improvement Program, translated into the local language. Senior nurses, selected early in the project as hand hygiene champions, delivered formal training sessions across departments. The campaign also incorporated creative elements to reinforce messaging—such as setting hand hygiene-related desktop wallpapers on hospital computers and providing small incentives (e.g., chocolates) to staff demonstrating high compliance. Unit-level tracking boards were introduced and maintained by charge nurses to foster visibility and accountability across teams.

### Nigeria antimicrobial stewardship pilot: gap analysis

The University of Benin Teaching Hospital (UBTH) is a 900-bed tertiary care, academic hospital without a formal antimicrobial stewardship program (AMP). In preparation for implementation of a surgical AMP, we identified local clinical microbiologists, infection control nurses, selected members of the surgical team, and members of the statistics unit of the hospital to lead the initiatives on the ground. An initial focus group was held with key stakeholders and local champions to fully understand the antibiotic climate at UBTH and discuss priorities for the AMP.

A baseline gap analysis was conducted using 35 patient records from 2 male surgical wards. Patients had a mean age of 48.9 ± 19.8 years. The most frequent diagnosis for admission and surgery was nephrolithiasis (22.9%), followed by prostate-related uropathies (20%), hernias (17.1%), and acute appendicitis (14.3%). Lesser common diagnoses included hemorrhoids, fistulas, and bowel obstructions. Urine and blood samples (97.2%) were most often collected and sent for culture with the two most common bacterial organisms cultured being Staphylococcus species and Klebsiella pneumoniae. The list of antibiotics prescribed is shown in Table 1. An overwhelming 81.2% of antibiotics were prescribed for prophylactic reasons whereas only 18.8% were prescribed for empirical reasons. Antibiotic indications were classified according to WHO and CDC surgical prophylaxis guidelines. Prophylactic use was defined as administration of antibiotics within 60 min prior to incision or shortly thereafter without signs of infection, whereas empirical use was defined as administration of antibiotics in response to clinical signs of infection prior to culture results.

**Table 1:** List of antibiotics prescribed for patients in the surveyed surgical wards at the university of Benin teaching hospital.

Antibiotics	Frequency* (*n* = 35)	Percent (%)
IV Ceftriaxone 1 g	13	37.1
IV Tandak 1.5 g (Ceftriaxone + Sulbactam) 1.5 g	11	31.4
Tab Marcfix 325 mg (Cefixime + Clavulanic acid)	10	28.6
Tab Cefuroxime 500 mg	6	17.1
Tab Levofloxacin 500 mg	5	14.3
Ciprofloxacin 500 mg	3	8.6
IV Flagyl	2	5.7
IV Amoxil 1.2 g	1	2.8
Enrofloxacin 500 mg	1	2.8
IV Meropenem 1 g	1	2.8
IV Gentamicin 80 mg	1	2.8
Cap Ampicillin + Cloxacillin 1 gm	1	2.8

With current data as described above, the proposed AMP package will be conducted using a multifaceted approach and the work is ongoing. The microbiology department will be leveraged to obtain culture results including those concerning antibiotic susceptibilities. Further, the most common surgical related infections and organisms will be collated and utilized within the antibiogram. We will use the free open-sourced software WHONET for creation of the antibiogram (https://whonet.org/). This antibiogram will be disseminated in the surgical wards and be utilized to guide antibiotic choices. Additionally, a seminar will be offered to the department of surgery that will highlight the formal AMP package as well as provide education on the importance of such programs. The key points of this seminar will include review of evidence for the utilization of antibiotic stewardship programs, the most common surgical procedures performed within UBTH and appropriate antibiotic prophylaxis for these procedures, most common postsurgical infections including speciation and treatment, introduction and utilization of the antibiogram, and an introduction to protocolized methods to measure antibiotic usage, manage microbiology consultations, and understand real time metrics of infection control. We anticipate that the seminar will be attended by surgeons, trainees, infection control staff including nurses, nursing aides and data collectors. Further, we hope that the seminar will incentivize a common communication channel between the microbiology and surgery departments and provide a forum for questions and discussions.

### Ethiopia trauma readiness pilot: curriculum review

Currently, Ethiopia has approximately 2,000 trained emergency medical technicians (EMTs) responsible for accompanying ambulance transports. However, this group comprises individuals with a limited educational background and without medical degrees. While there exists an established EMT curriculum supported by regional and national institutions, EMTs often face significant knowledge gaps regarding prehospital interventions to stabilize trauma patients. This lack of adequate training and knowledge hampers their ability to provide optimal care in emergency situations. Thus, a collaboration was created between the Ethiopian Ministry of Health and two non-governmental organizations (NGOs): Injury Prevention Initiative for Africa (IPIFA) and the G4 Alliance, with the goal of augmenting and formalizing the existing EMT curriculum in Ethiopia to promote task shifting and improve patient outcomes upon arrival at the hospital.

The most updated national EMT curriculum as set by the Ethiopian Ministry of Health was reviewed by a team of local and international experts during a multi-stakeholder workhop, including public health officials, international representatives from the G4 Alliance with prior experience in trauma systems development and Ethiopian surgeons from IPIFA who previously administered a bystander training course for all schoolteachers in the Addis Ababa region. The review process included comparison against WHO prehospital care guidelines, mapping of existing curriculum gaps, and consultation with frontline EMT instructors to identify areas where additional skills and protocols were most urgently needed. A total of 15 main topics were identified as lacking in the existing EMT training curriculum, including topics such as infection prevention and control, managing large-scale events, emergency vehicle operation, transport of emergency patients, obstetric emergencies, advanced life support, medication administration, creating high-performance teams, communication strategies, victim advocacy, individual and team development, establishing quality standards, organizational management, applying problem-solving techniques and tools, and implementing new technology. While this project has seen the slowest progress of the three pilots in this manuscript, it is undoubtedly also the most challenging and involves more stakeholder groups spread across governmental bodies and civil societies.

A curriculum is now being developed for each module listed above and a 15-session training course will be designed to empower first responders with the necessary skills and knowledge to stabilize trauma patients effectively. Through a train-the-trainer model, we aim to establish a sustainable system where trained EMTs can pass on their expertise to future cohorts, ultimately improving outcomes and laying the groundwork for national healthcare reform in trauma care.

## Discussion

The evolution of global surgery has shifted from direct delivery of care by foreign bodies to a heightened emphasis on building country-led initiatives and collaborative partnerships that have a long-lasting effect (4, 5). Our pilot initiatives conducted in Laos, Nigeria, and Ethiopia relied on local stakeholders to spearhead implementation projects that are relevant to their specific community. These initiatives have been **summarized in** Table 2, which provides a concise comparison of objectives, settings, stakeholders, frameworks, challenges, and progress across the three pilot sites. In Laos, we found that hand hygiene compliance was 43.9% and a perception survey highlighted that healthcare workers overestimate both their own and their co-workers' hand hygiene compliance. This gap may reflect workflow inefficiencies, inconsistent supply access, and limited accountability mechanisms. In Nigeria, we found that many patients housed in surgical wards were placed on antibiotics for non-empirical indications, due to limited culture availability, absent prescribing protocols, and poor integration between microbiology and surgical services. In Ethiopia, trauma experts reviewing the national EMT curriculum identified key gaps, stemming from underdeveloped EMS infrastructure and lack of formal training pathways. The urgent need for improvement to deliver effective high-volume surgical care to LMICs can be achieved through the creation, implementation, and evaluation of BPRs with the end goal of providing a high standard of quality surgical care worldwide (13).

**Table 2:** Summary of the three pilot implementation projects in Laos, Nigeria, and Ethiopia, including objectives, settings, stakeholders, guiding frameworks, outcome measures, challenges, and current status.

Category	Surgical Hand Hygiene Pilot	Surgical Antimicrobial Stewardship Pilot	Trauma Readiness Pilot
Objective/Aims	Improve hand hygiene adherence at Lao Friends' Hospital for Children and create an adaptable framework for other hospitals in Laos.	Develop a formal antimicrobial stewardship program (AMP) at University of Benin Teaching Hospital (UBTH) to optimize antibiotic use and reduce surgical infections.	Enhance training for Ethiopian EMTs to improve prehospital trauma care, reduce patient morbidity, and establish a sustainable task-shifting model for trauma management.
Country/Region	Lao PDR (Luang Prabang region)	Nigeria (Benin City)	Ethiopia
Healthcare Setting	Pediatric hospital (Lao Friends Hospital for Children)	Tertiary care, academic hospital (University of Benin Teaching Hospital)	Prehospital care (Ethiopian emergency medical transport system)
Beneficiaries/Stakeholders Involved	Local hospital staff, patients, G4 Alliance ISS-QSA working group	UBTH clinical microbiologists, infection control nurses, surgical team, statistics unit, patients	Ethiopian Ministry of Health, EMTs, NGOs (Injury Prevention Initiative for Africa and the G4 Alliance), Ethiopian surgeons, patients
Guiding Framework	WHO 5 Moments of Hand Hygiene, WHO Hand Hygiene Observation Form	WHONET for antibiogram creation, evidence-based AMP practices, antibiogram distribution in surgical wards	Ethiopian Ministry of Health's EMT curriculum, input from Ethiopian surgeons, train-the-trainer model
Metrics/Outcome Measures	Hand hygiene adherence rates, missed hand hygiene opportunities	Antibiotic usage and surgical site infection rates, adherence to AMP protocols	Number of EMTs trained, quality of prehospital trauma care, effectiveness of EMT interventions upon patient hospital arrival
Challenges Faced/Overcome	High rates of missed hand hygiene opportunities across hospital departments; overcoming awareness and adherence issues	Lack of formal AMP and baseline AMP data at UBTH; limited initial understanding of antibiotic use and infection control practices	Gaps in EMT training content, difficulty coordinating multiple stakeholder groups, broad range of necessary topics
Opportunities for Improving Quality of Surgical Care	Educational series, hospital-wide awareness campaign, and hand hygiene tracking boards for increased adherence and awareness	Implementation of AMP protocols, creation of antibiogram to guide antibiotic choices, education sessions for surgical staff on AMP principles and local antibiogram data	Task-shifting model for EMTs, focus on strengthening trauma response knowledge in infection control, emergency response, and advanced life support
Current Status/Planned Steps	Ongoing 12-month assessment; aim to inspire adoption of hand hygiene programs in other Laotian hospitals to improve patient morbidity and mortality.	Conduct AMP education seminar for surgery staff, improve communication between microbiology and surgery teams, disseminate antibiogram for guided antibiotic use	Curriculum development for 15 EMT training modules in progress; planned 15-session course to train EMTs on trauma stabilization techniques and introduce train-the-trainer model for sustainable EMT skill development

Pilot implementation projects are a crucial step in testing the feasibility and effectiveness of a larger initiative. They serve to identify potential causal mechanisms of change and facilitate an iterative process of refining intervention strategies to optimize their impact (14). Proctor et al. proposed a taxonomy of eight implementation outcomes: acceptability, adoption, appropriateness, feasibility, fidelity, implementation cost, penetration, and sustainability (15). Additionally, a recent paper by Henry et al. described integration, escalation, and maturity as key strategies for the implementation of surgical systems (7). However, several barriers exist to initiating pilot implementation projects, including lack of knowledgeable support, unaligned organizational culture, insufficient planning, limited generalizability of pilots, and the need for legislation/policy changes (16–18). Additionally, pilot studies that explore strategies to improve intervention implementation often require assessing changes across multiple levels, including individuals and organizational systems (19, 20). This is evident in the Ethiopia Trauma Readiness Pilot, which involved more stakeholder groups spread across governmental bodies and civil societies to facilitate improvement in the EMT curriculum. While these studies did not formally apply an implementation science framework, our findings align with key concepts from the field (15). Stakeholder engagement facilitated adoption and acceptability, while resource constraints and infrastructural limitations impacted feasibility and fidelity. The sustainability of these initiatives will depend on ongoing local leadership and integration into existing health systems. Future iterations could benefit from prospectively applying an implementation science framework to systematically assess barriers and facilitators.

Despite encountering numerous obstacles in the execution of these pilot projects, several factors bolstered their dependability and feasibility, laying a solid foundation for potential future implementation initiatives. Firstly, effective collaborative partnerships were established. The involvement of multiple stakeholders from different countries demonstrates a commitment to international collaboration in addressing healthcare challenges in LMICs. Secondly, local stakeholder engagement was prioritized. This ensures that interventions are tailored to the specific contexts and challenges faced by each healthcare center. This contrasts with the previous global surgery model of paternalistic interventionism, which can perpetuate systemic inequalities rather than effectively addressing them. In a recent paper by Krakaeur et al., it was articulated that a mutually beneficial experience can be achieved through collaborative efforts from both high-income and low-middle-income countries (21). Thirdly, inherent power dynamics were acknowledged and addressed by understanding their intrinsic nature. This study omitted external funding, which, while potentially posing a barrier, has the potential to mitigate financial power imbalances.

In discussing the findings, it's important to acknowledge the limitations of our study. Firstly, the sample size was restricted to three LMIC healthcare centres, potentially limiting the generalizability of our results to a broader context. Differences in facility type, resource availability, and cultural context are important to consider. For example, specialized centers such as pediatric hospitals or large academic institutions may have unique infrastructure and patient populations compared to smaller, rural facilities, while variations in financial and human resources, as well as local cultural norms, can influence both stakeholder engagement and the adoption of best practice recommendations. Secondly, as the projects are still ongoing, the presented results are preliminary, warranting longer-term follow-up to assess the sustainability and lasting impact of the interventions. Our reliance on descriptive analysis also presents a limitation, as it may offer limited insights compared to more rigorous analytical approaches. Moreover, while efforts were made to mitigate bias, the absence of explicit mention of bias control measures could impact the reliability and validity of our findings. It is important to note that some studies may have been affected by selection bias; institutions were chosen based on existing collaborations, which could limit generalizability. Measurement bias is another concern, as self-reported data in the Laos study and retrospective chart reviews in the Nigeria study may not fully capture reality. While both studies used standardized tools to minimize these issues, independent audits could further strengthen data reliability. Power dynamics may have also played a role in these studies, as hierarchical structures may have influenced data collection and stakeholder engagement. Thirdly, the absence of a formal implementation science framework, such as CFIR or Proctor's taxonomy, may limit the generalizability of our findings and ease of replication. While we chose a flexible, context-driven approach to adapt to each location's unique resources and challenges, a structured framework could have provided additional consistency and rigor. Future phases might benefit from incorporating a formal framework to enhance scalability and systematic monitoring of implementation outcomes. These limitations highlight the need for future research and improvement in promoting safe and quality surgical care in LMICs.

In conclusion, our study underscores the vital role of international collaboration in driving long-lasting initiatives to enhance surgical care in LMICs. By conducting comprehensive baseline needs assessments and gap analyses across three countries, we identified key pillars and metrics for the implementation of projects based on the 11 BPRs. These scalable pilot projects serve as a valuable framework for advancing the optimization and standardization of safe and quality surgical care in LMIC settings. However, these findings represent an early stage in the process, and further research is necessary to evaluate the long-term effectiveness and impact of these interventions. Moving forward, continued collaboration and implementation efforts guided by these findings will be essential in addressing the healthcare challenges faced by LMICs and improving surgical outcomes worldwide.

## Data Availability

The raw data supporting the conclusions of this article will be made available by the authors, without undue reservation.
